# Supplementary material for: Therapeutic Drug Monitoring of Ivacaftor, Lumacaftor, Tezacaftor, and Elexacaftor in Cystic Fibrosis: Where Are We Now?
Source: Pharmaceutics. 2022 Aug 11;14(8):1674. doi: 10.3390/pharmaceutics14081674 (PMC9412421; doi:10.3390/pharmaceutics14081674)
Supplement: Supplementary file 1 [file pharmaceutics-14-01674-s001.zip › pharmaceutics-1786435-supplementary.pdf]

Supplementary Materials

Therapeutic Drug Monitoring of Ivacaftor, Lumacaftor, Tezacaftor, and Elexacaftor in Cystic Fibrosis: Where Are We Now?

Eva Choong, Alain Sauty, Angela Koutsokera, Sylvain Blanchon, Pascal André and Laurent Decosterd

Table S1. Impact of Other Drugs on Elexacaftor, Tezacaftor and/or Ivacaftor [4].

Dose and Schedule		Effect on ELX, TEZ and/or IVA PK	Geometric Mean Ratio (90% CI) of ELX, TEZ, IVA No Effect = 1.0	
			AUC	C_{max}
Itraconazole 200 mg q12h on Day 1, followed by 200 mg qd	TEZ 25 mg qd + IVA 50 mg qd	↑ Tezacaftor<br>↑ Ivacaftor	4.02 (3.71, 4.63)<br>15.6 (13.4, 18.1)	2.83 (2.62, 3.07)<br>8.60 (7.41, 9.98)
Itraconazole 200 mg qd	ELX 20 mg + TEZ 50 mg single dose	↑ Elexacaftor<br>↑ Tezacaftor	2.83 (2.59, 3.10)<br>4.51 (3.85, 5.29)	1.05 (0.977, 1.13)<br>1.48 (1.33, 1.65)
Ketoconazole 400 mg qd	IVA 150 mg single dose	↑ Ivacaftor	8.45 (7.14, 10.0)	2.65 (2.21, 3.18)
Ciprofloxacin 750 mg q12h	TEZ 50 mg q12h + IVA 150 mg q12h	↔ Tezacaftor<br>↑ Ivacaftor *	1.08 (1.03, 1.13)<br>1.17 (1.06, 1.30)	1.05 (0.99, 1.11)<br>1.18 (1.06, 1.31)
Rifampin 600 mg qd	IVA 150 mg single dose	↓ Ivacaftor	0.114 (0.097, 0.136)	0.200 (0.168, 0.239
Fluconazole 400 mg single dose on Day 1, followed by 200 mg qd	IVA 150 mg q12h	↑ Ivacaftor	2.95 (2.27, 3.82)	2.47 (1.93, 3.17)

↑ = increase, ↓ = decrease, ↔ = no change. CI = Confidence interval; ELX= elexacaftor; TEZ = tezacaftor; IVA = ivacaftor; PK = Pharmacokinetics; * Effect is not clinically significant.

Table S2. PK parameters of ivacaftor and ivacaftor-lumacaftor standard therapy [14].

	IVA monotherapy healthy vs CF after 150mg	LUM/IVA	
		LUM	LUM/IVA
Mean (±SD) AUC	10.6 vs 5.26 µg·h/ml	n/a	198 ± 64.8 µg·h/ml LUM<br>3.66 ± 2.25 µg·h/ml IVA
Mean (±SD) C_{max}	0.768 vs 0.233 µg/ml	n/a	25.0 ± 7.96 µg/ml LUM<br>0.602 ± 0.304 µg/ml IVA
Time to steady state	3–5 days with accumulation ratio 2.2–2.9	After 7 days with accumulation ratio of 1.9	7 days (IVA when given with LUM)
